# Renal cancer-derived exosomes induce tumor immune tolerance by MDSCs-mediated antigen-specific immunosuppression

**DOI:** 10.1186/s12964-020-00611-z

**Published:** 2020-07-08

**Authors:** Yingying Gao, Haoyu Xu, Nan Li, Hexi Wang, Lei Ma, Shiyou Chen, Jiayu Liu, Yongbo Zheng, Yao Zhang

**Affiliations:** 1 Department of Laboratory Diagnosis, Chongqing Medical University, Chongqing, 408000 China; 2 Department of Laboratory Diagnosis, Jiamusi University, Jiamusi, 154000 Heilongjiang China; 3 Department of Urology, The First Affiliated Hospital of Chongqing Medical University, No. 1, medical college road, Yuzhong district, Chongqing, 408000 China; 4 Department of Laboratory Diagnosis, The First Affiliated Hospital of Jiamusi University, Jiamusi, 154000 Heilongjiang China

**Keywords:** RDEs, MDSCs, Antigen-specificity, Tumor immune escape, HSP70

## Abstract

**Backgound:**

Although Myeloid-derived suppressor cells (MDSCs) have a prominent ability to suppress the immune responses of T lymphocytes and propel tumor immune escape, a lack of profound systemic immunesuppression in tumor-bearing mice and tumor patients. The underlying mechanism of these remains unclear.

**Methods:**

For this purpose, renal cancer-derived exosomes (RDEs) were first labeled with PKH67 and been observed the internalization by MDSCs. Flow cytometry analysis showed the proportion and activity change of MDSCs in spleen and bone marrow induced by RDEs. Further, western blot experiments were used to verify triggered mechanism of MDSCs by RDEs. Finally, proliferation and cytotoxicity of cytotoxic T lymphocytes (CTLs) co-cultured with MDSCs in vitro and a series of experiments in vivo were performed to demonstrate the specific inhibitory effect of RDEs-induced MDSCs.

**Results:**

This study suggested that RDEs crucially contributed to presenting antigenic information, activating and driving specific immunosuppressive effect to MDSCs. HSP70, which is highly expressed in RDEs, initiate this process in a toll like receptor 2 (TLR2)-dependent manner. Importantly, RDEs-induced MDSCs could exert an antigen-specific immunosuppression effect on CTL and specific promote renal tumors-growth and immune escape in consequence.

**Conclusion:**

The immunosuppression mediated by MDSCs which is induced by RDEs is antigen-specific. HSP70, which is highly expressed in RDEs, plays a pivotal role in this process. Targeted abrogating the function of MDSCs, or eliminating the expression of HSP70 in exosomes, or blocking the crosstalk between them provides a new direction and theoretical support for future immunotherapy.

Video abstract

**Graphical abstract:**

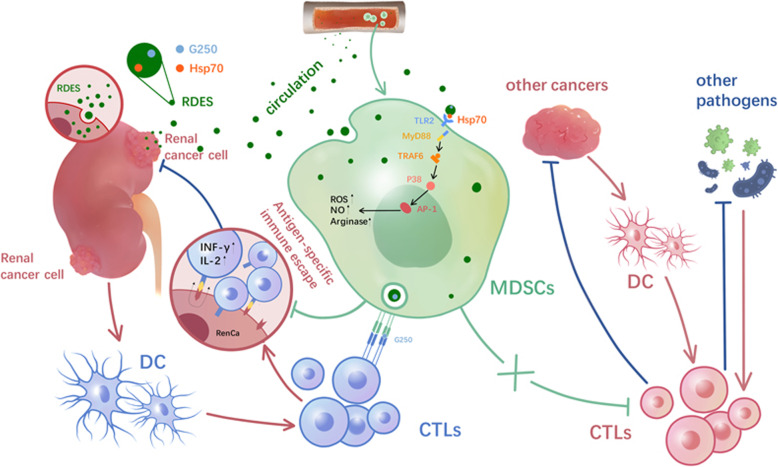

## Backgound

In spite of generally recognized that the essential of tumorigenesis is a result of genomic alteration, discoveries made over the past decades have suggested that an altered crosstalk between the tumor microenviroment and the host immune system may also provide growth advantages to tumor cells [1, 2]. Multiple studies have indicated that T lymphocyte anergic is one of the major mechanisms of tumor escape [3–5], however the mechanism is still ill certify.

Previous studies have reported that myeloid-derived suppressor cells (MDSCs) are a heterogeneous population of immature myeloid cells (IMCs) infiltrating the tumor microenvironment with potent tumor-associated T lymphocyte tolerance and immunosuppressive activity [4, 6, 7]. MDSCs are characterized by cell-surface markers CD11b^+^GR1^+^ in mice, while they are LIN^−^HLA-DR^−^CD33^+^ or CD11b^+^CD14^−^CD33^+^ in humans. In healthy individuals, they are the precursors of dendritic cells (DCs), macrophages and granulocytes [8, 9], while a obvious expansion was observed in bone marrow (BM) or tumor tissues of tumor-bearing mice or cancer patients [10–12]. However, in practice, tumor patients and tumor-bearing mice do not show systemic immunity dysfunction and can still have strong immune attack ability against other non-autoantigens.

Tumor cell-derived exosomes (TDEs) are multivesicular bodies and secreted by tumor cells, with diameters ranging from 30 to 100 nm. Our previous researches showed a lot of basic research on renal cancer-derived exosomes (RDEs) [13, 14] and found that it carried a set of tumor-associated antigens, and immune modulation molecules such as G250, heat shock protein (HSP), tetraspanins, major histocompatibility complex molecules (MHC) I and II, adhesive molecule ICAM-1, et al. Recent study also showed TDEs dictated the amplification of MDSCs [15]. However, the triggered mechanisms of activation have not been fully addressed.

Myeloid differentiation primary-response gene 88 (MyD88), an important cytoplasmic adaptor molecule for integrating and transducing the signals triggered by all Toll-like receptors (TLRs) family except TLR3, has been reported inducing MDSC expansion in sepsis [16]. While HSP70, overexpressed in RDEs, is an important endogenous ligand of TLRs [17].

Based on these, this study clarified our speculation that the immunosuppression of T lymphocyte drove by MDSCs is antigen-specific nature. RDEs and embedded HSP70 is actually responsible for MDSCs amplification, activation and induce renal tumor immune escape through a comprehensive of studies in vivo and vitro. Importantly, our findings provide a new idea for immunnotherapy and find a new break for targeted therapy of renal cell carcinoma, which has scientific exploration and clinical application prospect.

## Materials and methods

### Mice

Six-to-eight-week-old BALB/c mice, weighing 16–22 g, were purchased from and house in the Animal Experimental Center of Chongqing Medical University (Chongqing, China). All mice were fed under pathogen-free conditions with laminar air flow. All experimental manipulations were approved by the Ethics Committee of The First Affiliated Hospital of Chongqing Medical University.

### Cell lines and culture

The mouse renal adenocarcinoma cell line, RenCa, breast cancer cell line, 4 T1, and colon cancer cell line, CT26, were purchased from Shanghai Cell Bank. Cells were cultured in RPMI-1640 medium (Thermo Fisher Scientific, Inc. Waltham, MA, USA) with 10% fetal bovine serum (FBS) (Thermo Fisher Scientific, Inc.) and 1% penicillin and streptomycin (Beyotime Institute of Biotechnology, ShangHai, China) in 37 °C incubator with 5% CO_2_.

### Exosome isolation, identification

RDEs were isolated from conditioned medium collected from RenCa cells with or without HSP70 knockdown. Several centrifugation and filtration steps were used as previously described [13]. The specific operation steps are as follows: Culture supernatants (100 ml) were collected and sequentially centrifuged (4 °C) at 300×g for 10 min, 800×g for 30 min and 10,000×g for 30 min. The clarified supernatant was concentrated by centrifugation at 1000×g for 30 min in a prerinsed 100 kDa MWCO Centrifugal Filter Device and the concentrated exosomes were collected and resuspended in 20 ml PBS. The ultracentrifuge supernatant was underplayed with 30% sucrose/D_2_O density cushion, followed by ultracentrifuging at 100,000×g for 60 min. At the bottom, the cushion was collected and diluted in 10 ml of PBS. The exosomes were further concentrated by centrifuging for 30 min at 1000×g inprerinsed 100 kDa MWCO Amicon ultra-15 to a volume of about 3 ml. Membrane filter (0.22 μm) was used, after sterilization the exosome was stored at − 80 °C. After protein concentration was determined using BCA method (Beyotime Institute of Biotechnology), the morphological characteristics of RDEs were identified by transmission electron microscope (TEM) (JEM-2010, Jeol Ltd., Tokyo, Japan).

### Isolated MDSCs from spleen or BM and analyzed by flow cytometry

Single-cell suspensions without erythrocyte were prepared from mice spleens or BM of BALB/c on different time point after treatment. Gr-1^high^Ly-6G^+^ and Gr-1^dim^Ly-6G^−^ cells were respectively selected by being indirectly magnetically labeled with anti-Ly-6G-Biotin and anti-Biotin Microbeads (Miltenyi Biotech, Bergisch Gladbach, Germany) and anti-Gr-1-Biotin and Streptavidin Microbeads (Miltenyi Biotech). After the above two parts were collected together, MDSCs were stained with anti-Gr-1-FITC and anti-CD11b-PE antibodies (Biolegend, San Diego, CA, USA) and the proportions were analyzed by flow cytometry (Beckman Coulter, Pasadena, CA, USA). Then 10 ng / ml GM-CSF (PeproTech, Shanghai, China) and 2 ng / ml IL-6 (PeproTech) were supplemented to the medium and cultured at 37 °C with 5% CO_2_.

### Uptake of exosomes by MDSCs

To perform uptake experiments, RDEs were labeled with PKH67 according to operating procedure (MINI67, Sigma-Aldrich, Merck KGaA, Darmstadt, Germany). Briefly, 200 μg RDEs were resuspended in 2 ml PBS with 4 μl PKH67 for 5 min in 37 °C. The final concentration of PKH67 is dye 2 × 10^− 6^ mol/L.

Next, 2 ml 1%BSA was added to terminate the staining for 1 min, and the volume of the mixture was supplied to 20 ml with 1% BSA. Then, the mixture was centrifuged at 120,000 g for 1 h, repeatedly three times. The precipitation was the PKH67-labeled RDES. Twenty microgram of the PKH67-stained RDEs were co-cultured with 2 × 10^5^ MDSCs, and cells were harvested at 0 h, 6 h or 12 h. At the same time, the 100 μg of the PKH67-stained RDEs were injected intravenously to every mouse, and the splenetic MDSCs were extracted at 0 h, 24 h or 48 h. All of these MDSCs were fixed, dyed nuclei and visualized with confocal microscopy.

### ROS detection, arginase activity and NO production

The level of ROS production was measured using the oxidation-sensitive dye 2′, 7′-dichlorodihydrofluorescein diacetate (DCFDA) (Thermo Fisher Scientific, Inc.). MDSCs were simultaneously incubated with 30 ng/ml Phrobol-12-myristate-13-acetate (PMA) (Sigma-Aldrich) and 4 μmol/L DCFDA for 30 min at 37 °C. Median Fluorescence Intensity (MFI) was used to mark the production of ROS in 535 nm by flow cytometry.

Arginase activity test using Arginase Activity Assay Kit (MAK112, Sigma-Aldrich) performed with the manufacturer’s protocol.

To detect NO, Cell culture supernatant mixed with the same volume of Griess reagent for 10 min at 25 °C. Absorbance was measured at 550 nm, and the concentrations of nitrite were calculated according to the standard curve.

### Western blot

RenCa cells were transfected with HSP70 knockdown lentiviral particles and negative control (mock) (Shanghai GenePharma Co., Ltd., Shanghai, China). The expression of HSP70 in RDEs were checked by western blot. Then, MDSCs were co-cultured with RDEs^shRNA mock^ or RDEs^shHSP70^, or TLR-2 inhibitor (C29, 363,600–92-4, MCE). The protein extraction and Western blot analysis were performed as described previously [18]. The membranes were incubatedwith primary antibodies overnight at 4 °C, then sequentiallyincubated with secondary antibodies for 2 h at room temperature. The intensity analyses were quantified using Image-Pro Plus 6.0 software. The primary antibodies and secondary antibodies incubated were as follows: The rabbit anti-mouse p38 (#8690), p-p38 (#4511), AP-1 (#9164), Alix (#92880), TLR-2 (#13744), MyD88 (#4283) were obtained from Cell Siganling Technology (CST). TSG101 (sc-7964), CD63 (sc-5275), GM130 (sc-55,590), TRAF6 (sc-8409) were obtained from Santa Cruz Biotechnoligy. HSP70 (ab181606), G250 (ab184006), horseradish peroxidase (HRP)-conjugated goat anti-rabbit secondary antibodies (ab6940), horseradish peroxidase (HRP)-conjugated goat anti-mouse secondary antibodies (ab97040) were obtained from Abcam (Cambridge, MA, USA).

### Preparation of tumor cell lysates

The tumor cell lysates were obtained from three types of tumor cell lines according to the previous methods [19]. By 5 cycles of freeze and thaw, Lysates were centrifugated at 800×g for 30 min, and the supernatants were filtered with a 0.22 μm filter and stored at − 20 °C.

### Maturation of DCs pulsed by tumor cell lysates

BM-derived DCs were isolated from mouse BM according to a previously described [20], with a certain modification. Erythrocyte-depleted mouse BM cells were cultured in complete medium supplemented with GM-CSF (10 ng/ml) and IL-4 (10 ng/ml) (PeproTech, Rocky Hill, NJ). On day 7, tumor cell lysates were incubated with purified DCs at a ratio of 3:1. The phenotypic profile of DCs was detected by flow cytometry.

### Cytotoxic assays

To confirm cytotoxic T lymphocytes (CTLs), the BM-derived DCs, pulsed by tumor cell lysates, were injected subcutaneously into syngeneic mice. Unpulsed DCs and PBS were used as paralleled experimentations. As the protocol described previously [19], 14 days after injection, CD8+ T lymphocytes were sorted from mice splenocyte using CD8 microBeads (Miltenyi Biotech) [21]. Lactate dehydrogenase (LDH) release assay using CytoTox96 Non-Radioactive Cytotoxicity Assay Kit (Promega Biological Products, Ltd., Shanghai, China) was used to measure the cytolysis rate elicited by effector T lymphocytes against different tumor cells. Specific lysis (%) was calculated based on the equation: (Experimental LDH release − effector cells − target spontaneous LDH release) / (target maximum LDH release) × 100.

### Inhibitory effect of MDSCs on CD8+ T lymphocytes proliferation

1 × 10^7^ CTLs, derived from splenic CD8+ T lymphocytes stimulated by three different tumor cells lysates-pulsed DCs, were labeled with 2.5 μM carboxyfluoresceindiacetate succinimidylester (CFSE, Macklin Biochemical Co., Ltd., Shanghai, China) at 25 °C for 10 min in dark. Then different tumor cells antigen-stimulating CTLs were co-cultured with MDSCs^RDE^ or MDSCs^PBS^ with a ratio of 5:1, PBS with same volume of were used as controls. After 24 h, the proliferation of CTLs was analyzed by flow cytometry.

### Tumor growth assays

Three different kinds of tumor model were established by subcutaneous injection with RenCa, 4 T1 or CT26 tumor cells (3 × 10^6^ cell per mouse), respectively. Each tumor model was intravenously with MDSCs^RED^ or MDSCs^PBS^ (1 × 10^6^ cells in 200 μl PBS) for 3 times a week. Same volume PBS was intravenously at the same interval as control group. The tumor size was assessed every 2 days. After 3 weeks, the mice were sacrificed by deep inhalation anesthesia (2–3% isoflurane) and local analgesia (oxybuprocaine hydrochloride). Tumor tissues were isolated for comparing the size and performing histochemical experiments. Calculation of the formula V = π/6 x L × W (L: length; W: width).

### Statistical analysis

All data were reported as Mean ± SD and repeated ≥3 times independently. One-way analysis of variance (ANOVA) and two-way ANOVA were used evaluated the significant difference among groups using SPSS 21.0 (IBM Corp., Armonk, NY, USA) and GraphPad Prism software version 5 (GraphPad Software, Inc., La Jolla, CA, USA). A value of *P* < 0.05 was considered as statistically difference.

## Results

### Tumor specific-antigen and HSP70 were enriched in RDEs

**Fig. 1:**
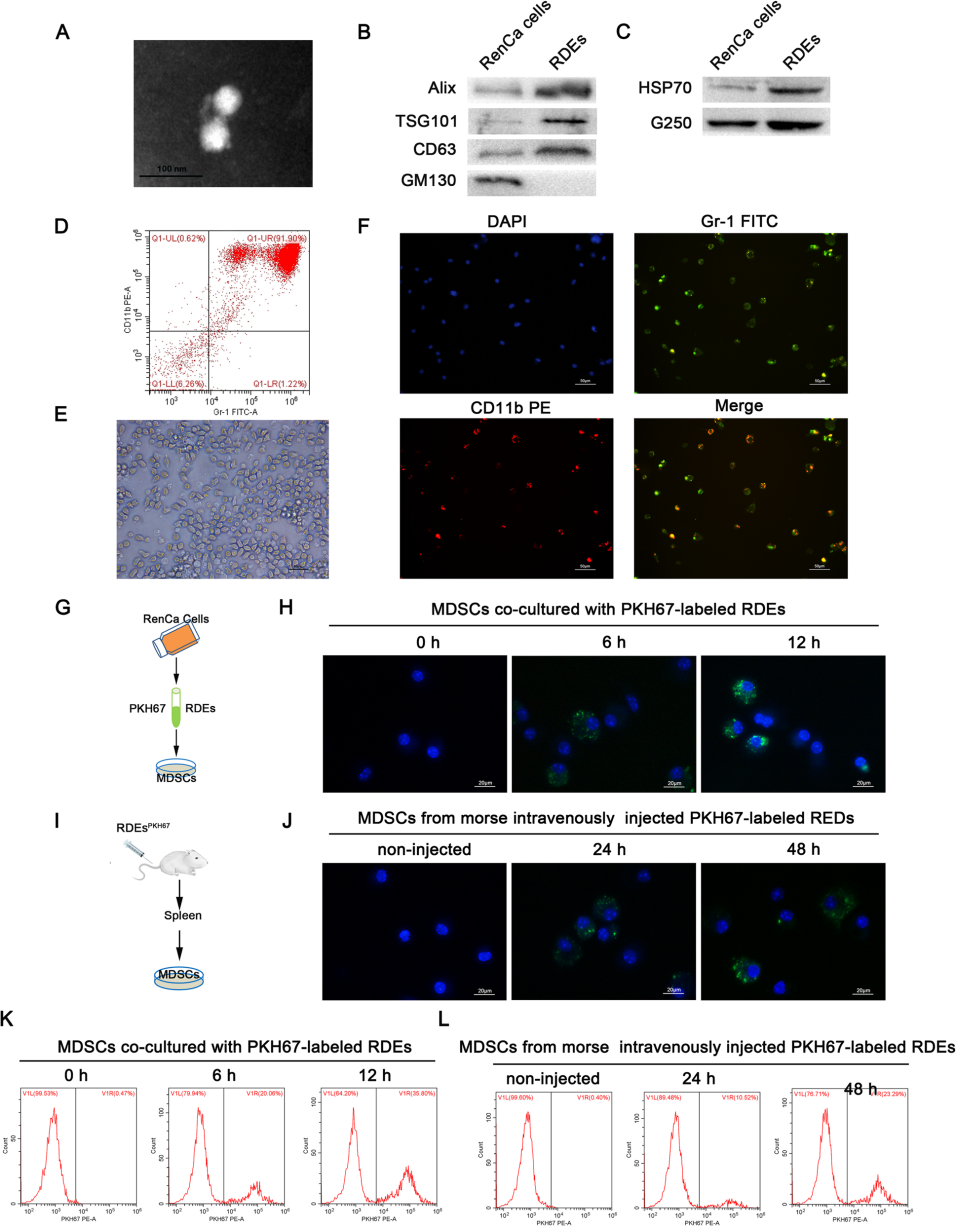
RDEs enriched with tumor specific-antigen and HSP70 could captured by MDSCs. **a** Typical morphologies and sizes of RDEs were identified by transmission electron microscopy (TEM). **b** Western blot was used for detecting the expression of exosomal biomarkers and cellular protein or **c** Renal-specific antigens (G250) and immuno-modulators (HSP70) in RenCa cell and RDEs. Thirty microgram of total protein was loaded for RenCa cell and RDEs, respectively. **d** The separation rate of MDSCs was verified by flow cytometry. The cellular morphology and characterization of MDSCs were observed by microscope (**e**) and immunofluorescence assay analysis (**f**). **g**, **i** Schematic representation of the experiment in vitro and in vivo. **h** Representative phagocytosis of PKH67-labelled RDEs by MDSCs at 0 h, 6 h or 12 h in vitro. **j** Phagocytosis of RDEs by splenetic MDSCs in vivo at non-injected and 24 h or 48 h after intravenous injection with PKH67-labelled RDEs. The image was observed by confocal microscopy. Green, PKH67-labelled RDEs; Blue, DAPI. **k** Representative graph of fluorescence of MDSCs detected with flow cytometry as described in (**h**). **l** Representative graph of fluorescence of MDSCs from mouse spleen after tail intravenously injected PKH67-labeled RDEs as described in (**j**). All experiments were repeated at least three times and three mice were in each group

To test the feasibility of RDEs as a source of specific-antigens for antitumor immunity in renal carcinoma, RDEs were first isolated from the serum free culture supernatants of RenCa cells. They exhibited spherical membrane-bound vesicles surrounded by the two-layer lipid membrane with a mean diameter of 50.1 ± 23.2 nm and most diameters between 30 and 80 nm by TEM (Fig. 1a). The protein yield of RDEs were about 0.35–0.5 μg per 1 × 10^6^ tumor cells in 24 h, as other tumor cells [22]. As expected, the RDEs sample was positive for exosomes biomarker proteins including transmembrane protein CD63, TSG101 and Alix [23], but negative for cir-Golgi marker GM130, which was only celluar protein (Fig. 1b). Further investigation revealed enriched renal carcinoma specific antigen G250 and immuno-modulators HSP70 was expressed in RDEs compared with their parental cells (Fig. 1c), which suggested that RDEs have the ability to present tumor specific-antigens and deliver antigen information to interacting target cells.

### RDEs carrying tumor antigens are captured by MDSCs

In this study, immunomagnetic beads were used to isolate MDSCs and the flow cytometry verified the separation rate of positive cells were more than 90% (Fig. 1d). After cellular morphology was observed by microscope (Fig. 1e), immunofluorescence assay was used to identify the Gr-1 and CD11b double-positive cells (Fig. 1f). This verified their validity to be used in the later functional experiments.

To examine whether RDEs can be uptake by MDSCs in vitro, the RDEs, labeled with PKH67, were co-cultured with MDSCs (Fig. 1g). The internalization of PKH67-labelled RDEs by MDSCs was perinuclear and punctuate in appearance observed by fluorescence microscope at 6 h or 12 h (Fig. 1h). Next, to evaluate the ability of MDSCs to uptake RDEs in vivo, we isolated the splenic MDSCs at 24 h or 48 h after tail intravenously injected PKH67-labeled RDEs (Fig. 1i). Fluorescence signal was traced in these MDSCs using fluorescence microscope (Fig. 1j). Consistently, flow cytometry analysis showed an inceased fluorescence of MDSCs after the addition of PKH67-labeled RDEs in vitro, or after labeled RDEs were intravenously injected in vivo (Fig. 1k-l). The results revealed that the RDEs could be captured by MDSCs. In the same way, MDSCs might capture tumor antigen information.

### RDEs drived the expansion and activation of MDSCs

**Fig. 2:**
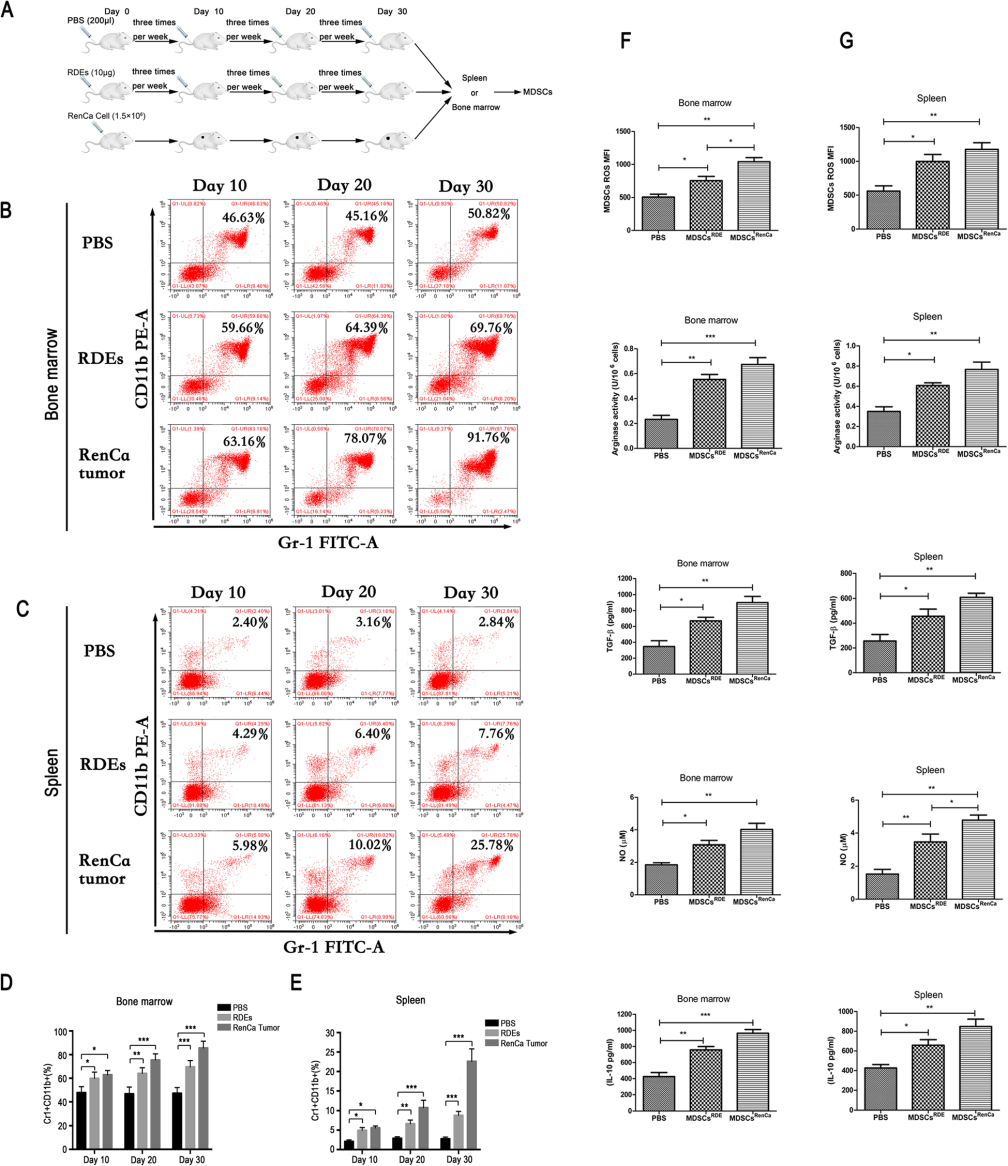
RDEs drived the expansion and activation of MDSCs in vivo. **a** Experimental scheme of induced and isolated MDSCs. Representative flow cytometry experiment showed the percentage of cells expressing Gr-1+ and CD11b + markers in BM cells (**b**) and speen (**c**) at days 10, 20 and 30 with the treat of PBS, RDEs or RenCa cells, and the statistical resultes were showed in (**d** and **e**). The production of ROS, NO and arginase activity, as well as IL-10 and TGF-β expression were measured in MDSCs isolated from BM (**f**) and spleen (**g**). All experiments were repeated at least three times and three mice were in each group

To further examine whether exosome have ability to induce the expansion and activation of MDSCs, BALB/c mice model was intravenously injected RDEs (10 μg in 200 μl/mouse) or PBS (200 μl/mouse) respectively three times per week for 30 days, and subcutaneously injected RenCa cells (1.5 × 10^6^/mouse) as a positive control. After 7 days, Renca cells formed small transplanted tumors under the skin. Then spleen and BM cells were isolated at 10, 20, 30 days respectively to examine the expansion of MDSCs (Fig. 2a). Flow cytometry analysis showed compared with control group treated with PBS, RDEs group and RenCa cells group showed significantly increased the expansion of Gr-1^+^CD11b^+^ populations in BM cells at days 10, and more obviously increased in days 20 and days 30. Consistent results were obtained in spleen cells (Fig. 2b-e). Taken together, these results indicated that RDEs have the potential to induce MDSCs expansion in BM and spleen.

Historically, the activity of MDSCs in a pathological context is associated with the upregulated expression of immune suppressive factors such as arginase 1, and increased production of NO, and reactive oxygen species (ROS) [4]. Thus, compared with MDSCs isolated from BM of PBS group, MDSCs induced from RDEs produced more ROS and NO and exhibited stronger arginase activity, just as these factors were activated in Rence cells-induced MDSCs. The secretion of other factors which were involved in immunosuppressive functions, such as IL-10 and TGF-β, were also increased in RDEs-induced MDSCs group (Fig. 2f). The activity of MDSCs in spleen exhibited the similar results (Fig. 2g). These results suggested RDEs were involved in the induction of MDSCs amplification and activation. However, the inducing mechanism needs to be explored further.

### The activated of MDSCs induced by HSP70 expressed in RDEs

**Fig. 3:**
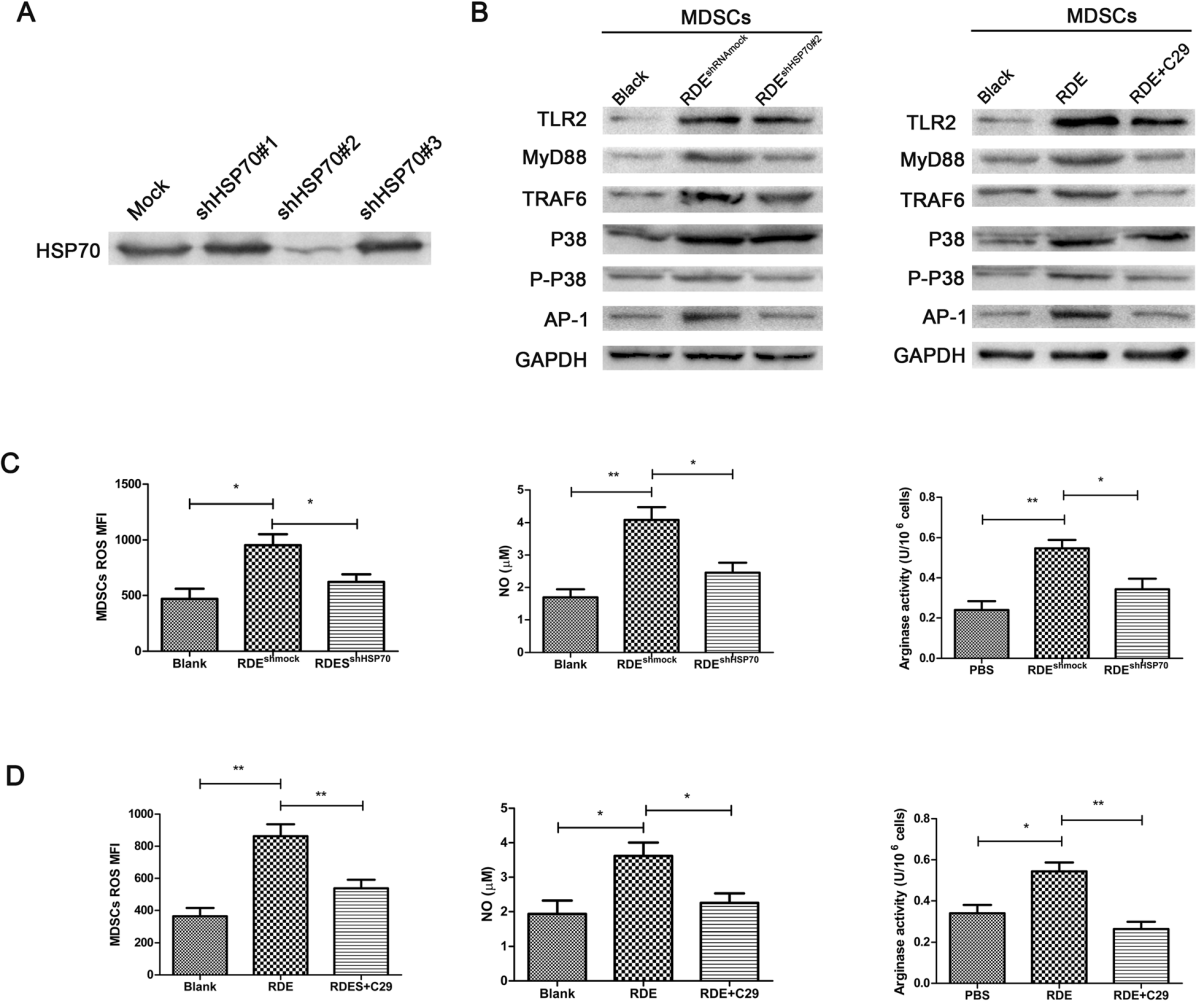
The activation of MDSCs is triggered by RDE-associated HSP70 in TLR2-dependent signal pathway. **a** RDEs were isolated from conditioned medium after transfecting of RenCa cells with HSP70 knockdown Particles (shHSP70) or negative control particles (shRNA mock). Same amount of protein of RDEs were used in different groups and HSP70 expression in RDEs was checked by western blot. Next, we chose #2 lentivirus particles of knockdown HSP70 to be used in the later experiments. **b** Western blot of TLR2 and its downstream factors expression from MDSCs co-culturing with RDEs with or without shHSP70, or RDEs with or without C29, a inhibitor of TLR2. **c**, **d** ROS or NO production and arginase activity were measured after MDSCs were treatment described in (**b**). GAPDH served as control. All experiments were repeated at least three times

To investigate whether HSP70 plays a significant role in activation of MDSCs, a series of HSP70 knockdown experiments were used in Renca cells. After the exosomes from Renca cells were isolated, HSP70 expression in RDEs were checked by western blot. ShHSP70#2 markedly reduced the expression of HSP70 in RDEs and was used in later experiment (Fig. 3a). Subsequently, MDSCs were co-cultured with RDEs^shHSP70^ or RDEs^shRNAmock^ in vitro. The results showed that RDEs could upregulate the expression of TLR2 and its downstream factors, such as MyD88, TRAF6, P38 and AP-1 in MDSCs (Fig. 3b). And with the induction of RDEs, MDSCs produced most ROS and NO and exhibited stronger arginase activity compared with the MDSCs primitive culture group (Fig. 3c). While, the expression of MyD88 and its downstream factors were blunted by HSP70 knockdown (Fig. 3b). Consistently, the activity of MDSCs is also significantly down-regulated (Fig. 3c).

In order to further characterize the activation mechanism of MDSCs, we treated the cells with TLR-2 inhibitor. The results showed that, TLR2 inhibitor can also reversed the expression level of factors in TLR/MyD88 signaling pathway (Fig. 3b) and the production of ROS and NO or arginase activity in MDSCs, which were up-regulated by RDEs (Fig. 3d). Taken together, these results suggested that the proliferation and activation of MDSCs were mediated via the binding of HSP70 in RDEs to TLR2 expressed on MDSCs.

### T lymphocytes immune responses were launched by the maturation of DCs

**Fig. 4:**
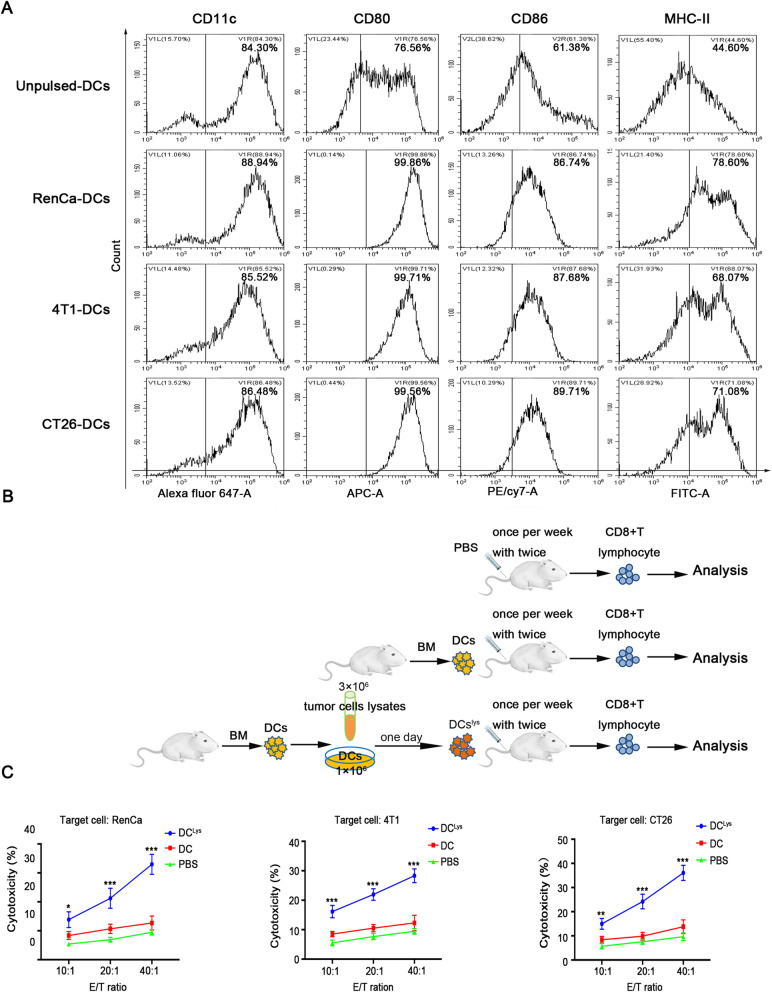
T lymphocytes cytotoxicity were launched by the maturation of DCs. **a** Flow cytometry for analyzing levels of surface proteins in DCs pulsed with RenCa cell lysate, 4 T1 cell lysate, CT26 cell lysate or PBS (unpulsed control). **b** Experimental scheme was used for description the cytotoxicity of CD8 + T lymphocytes induced by DCs. **c** Cytolysis assay for CD8 + T lymphocytes activated by DCs^lys^ or DCs against autologous tumor cells at different E: T (effector: target) ratios. PBS was used as negative control. The data are representative of three independent experiments and expressed as the mean ± SD (*P < 0.05). DCs^lys^, the group of tumor cell lysate-pulsed DCs; DCs, the group of unpulsed DCs; PBS, the group of PBS-stimulated CD8 + T cells. All experiments were repeated at least three times and three mice were in each group

DCs act as a bridge in the immune system. Matured DCs play a crucial role in capturing, processing and presenting tumor-specific antigens to T lymphocytes and initiate T lymphocyte proliferation and differentiation into helper and effector cells. To investigate these, we first stimulated DCs differentiation and maturation using freezing-thawing tumor cells lysates. Compared with unpulsed group, tumor lysates of RenCa, 4 T1 and CT26 caused remarkable up-regulation of CD80, CD86 and MHC-II expression in DCs (Fig. 4a), which suggested the maturation of DCs could induced by tumor cells lystates.

To examine if matured DCs could drived T lymphocytes to trigger cytotoxicity against autologous tumor cells, CD8 + T lymphocytes acted as effector cells, extracted from the spleen of mice, were hatched with tumor cells lysates-pulsed DCs, unpulsed DCs or PBS (Fig. 4b), while autologous tumor cells acted as target cells. Coresponding E/T (effector/target) ratios were shown in Fig. 4c. The results suggested RenCa cell lysate-pulsed DCs stimulated CD8 + T lymphocytes (CTLs^RenCa^) displayed potent cytotoxic ability against RenCa cells at all E/T ratios compared with unpulsed DCs or PBS stimulated. Similar results can be observed at 4 T1 or CT26 cell lysate-pulsed DC-stimulated CD8 + T (CTLs^4T1^or CTLs^CT26^). This indicates that matured DCs provokes T lymphocytes-dependent antitumor immunity.

### RDEs-induced MDSCs suppressed the cytotoxic effect of CD8 + T lymphocyte only on renal cancer cells

**Fig. 5:**
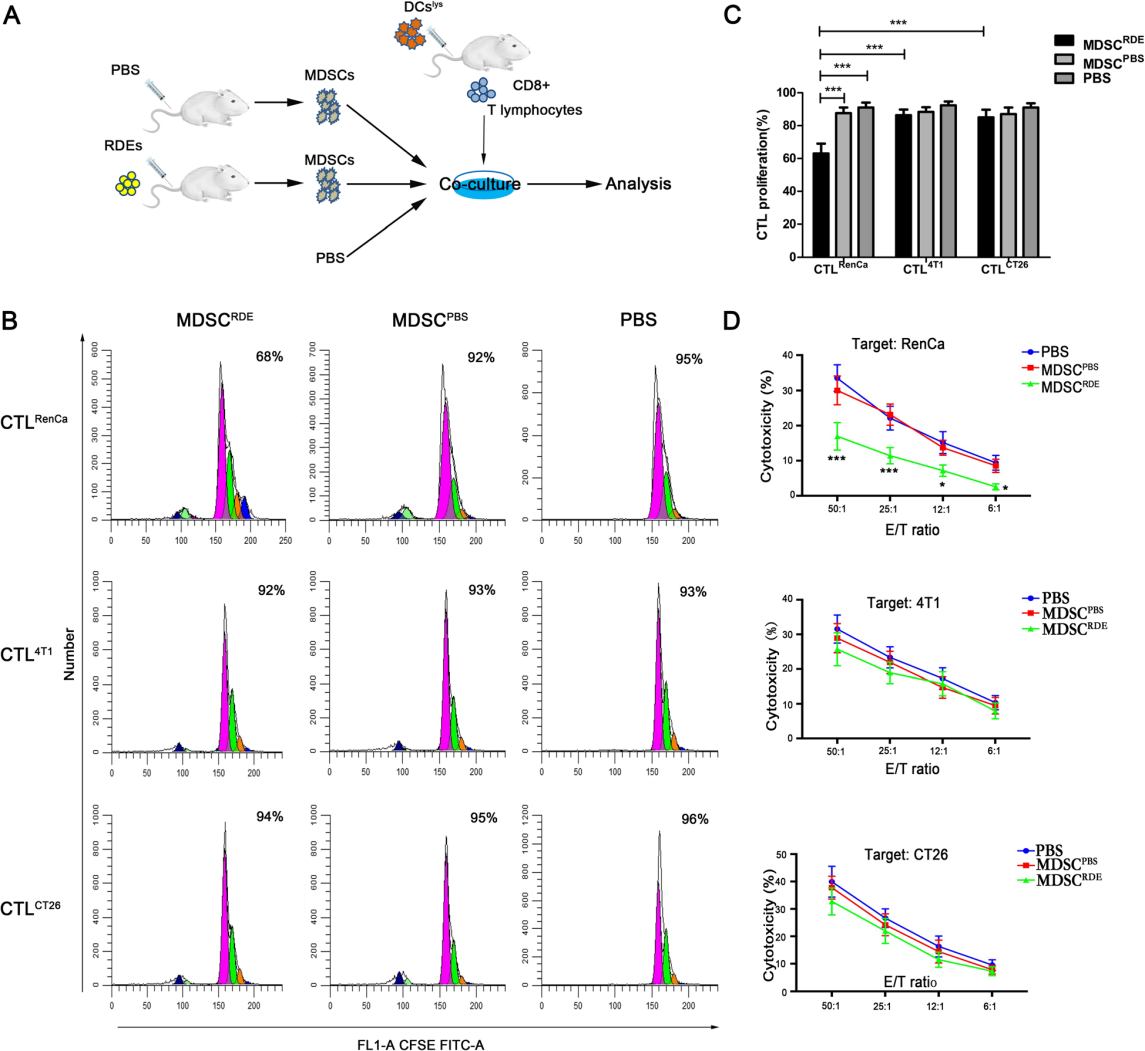
MDSCs specific-suppressed antitumor immunity of CTLs. **a** Experimental scheme. **b**-**c** Flow cytometry for analyzing the proliferation percentage of CTLs affect by MDSCs^RDE^ or MDSCs^PBS^, PBS was used as negative control. **d** Comparison of cytotoxic effect of different sourced CTL treated with MDSCs^RDE^ or MDSCs^PBS^ against autologous tumor cells, respectively. CTL^RenCa^, the RenCa cell lysate-pulsed DC-stimulated CD8 + T cells; CTL^4T1^, the 4 T1 cell lysate-pulsed DC-stimulated CD8 + T cells; CTL^CT26^, the CT26 cell lysate-pulsed DC-stimulated CD8 + T cells; MDSCs^RDE^, RDE induced-MDSCs; MDSCs^PBS^, PBS induced-MDSCs. All experiments were repeated at least three times and three mice were in each group

To address our speculation that the immunosuppression effect of CTLs drived by RDEs-induced MDSCs is antigen-specific, CTLs obtained from the splenocytes of BALB/c mice stimulated by renal, breast or colon tumor cell lysates-pulsed DCs respectively. Three different sources of CTLs loaded with fluorescent dye CFSE were co-culture respectively with MDSCs induced by RDEs or not in vitro, PBS-treatment were used as negative controls (Fig. 5a).

Flow cytometry analysis showed MDSCs^RDE^ could only significantly reduced the proliferation percentage of CTLs^RenCa^ compared with CTLs from other two sources, while there was no significant difference in the influence of MDSCs^PBS^ or PBS (Fig. 5b, c). Consistently, MDSCs^RDE^ could significantly inhibited cytotoxicity of CTL^RenCa^ compared with MDSCs^PBS^ or PBS, but no obvious difference were observed on CTL^4T1^ and CTL^CT26^ (Fig. 5d). All the results indicated that TDEs-induced MDSCs exerted an obvious inhibitory effect on the proliferation and activation of CTLs, and the inhibitory effect is homologous specific.

### MDSCs activated by RDEs accelerated homeograft tumor growth through immunosuppression of CTL

**Fig. 6:**
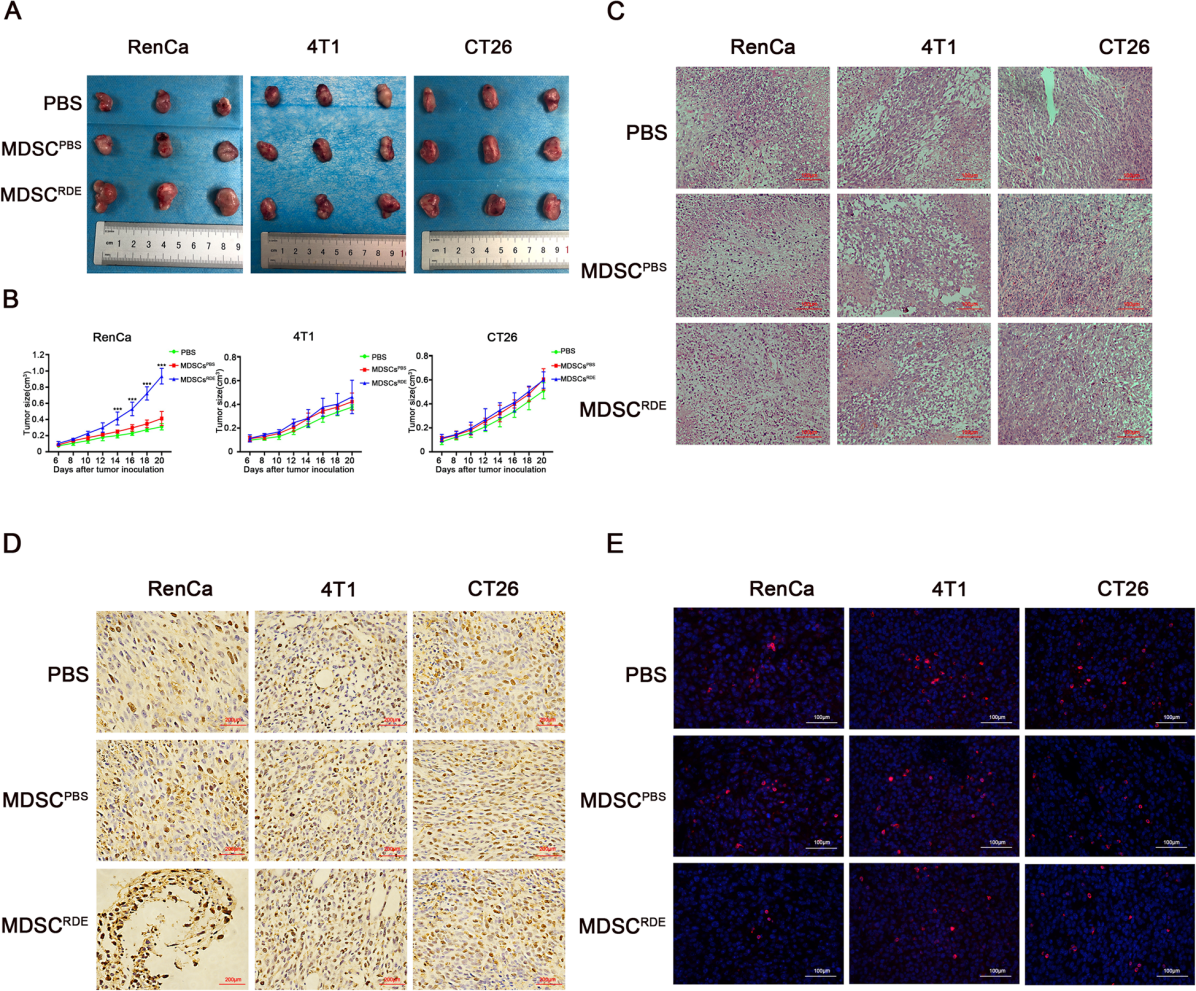
MDSCs activated by RDEs accelerated homologous tumor growth through immunosuppression of CTL. Three different tumor models were established using BALB/c mice subcutaneously injected with Renca, 4 T1 and CT26 cells. Each model was divided into three groups, treated with MDSCs^RED^, MDSCs^PBS^ or PBS, respectively. **a** The tumor was stripped and showed after the mice were sacrificed on days 20 after subcutaneous tumor cells. **b** Analysis and compare of tumor volume every 2 days in different treatment. **c** Representative haematoxylin and eosin (H&E) staining of different group tumor model. **d** Representative Ki-67 immunohistochemical staining of different group (brown, Ki-67; blue, nuclei). **e**. CTLs infiltration in tumor tissues of various tumor models with different treatment by ISTS. Fluorescence images of tissue sections stained with CD8+ T in red and cell nuclei were labeled with DAPI (blue). All experiments were repeated at least three times and three mice were in each group

To further corroborate the antigen-specific immunosuppressive activity and tumor growth-promoting effect of MDSCs in vivo, we established three different tumor models of BALB/c mice using Renca, 4T1and CT26 cells injected subcutaneously. Next, MDSCs^PBS^ or MDSCs^RDE^ was respectively injected intravenously in every tumor model, PBS was performed as negative control. The tumor size was measured at different time points. As the tumor microenvironment was consistent in all tumor models. Interestingly, supplementary MDSC^RDE^ treatment significantly increased renal tumor growth compared to MDSC^PBS^-treated group (Fig. 6a-b). Consistently, a major increased in Ki67 positive cells were impacted by MDSCs^RDE^. But this significant difference in tumor growth and Ki67 positive cells between the MDSCs^RDE^ groups and the MDSCs^PBS^ groups was not observed in 4 T1 or CT26 tumor-bearing mice (Fig. 6c-d). Next, In situ tetramer staining (ISTS) was used to show the presence of tumor-infiltrating CTLs in tumor-bearing mice, which showed the amount of CTLs at tumor sites were significantly decreased in renal tumor model when mice were treated with MDSCs^RDE^ compared to other tumor model (Fig. 6e). Taken together, these data supported the idea that MDSCs activated by TDEs can support the tumor growth through immunosuppression of CTL, but this growth promotion effect can only be targeted at homologous tumor.

## Discussion

Defective T lymphocyte function induced by MDSCs represents one of critical factors in tumor progression [24, 25]. In our study, a significant increase in MDSCs proportion and activation was observed in spleen and BM of tumor-bearing mice or normal mice following immunization with RDEs. Other studies also support our result. Accumulating evidences have shown, in different tumor model mice splenocytes, up to 20–40% of nucleated cells are MDSCs, while 2–4% in normal mice. MDSCs are also found in tumor tissues of tumor-bearing mice or tumor patients peripheral blood [9, 26]. However, the suppressive activity of MDSCs depend not only on the expansion of their proportion but also on the induction of their activation. RDEs, in our study, were also verified to increase the production of ROS, NO and strengthen the arginase activity in MDSCs. The direct role for these factors in the inhibition function of T lymphocyte is well established, although the immunosuppressive manner of MDSCs in these contexts is antigen-nonspecific. They could induct T lymphocyte apoptosis through blocking the activation of JAK3, STAT5 transcription factor, inhibiting the expression of MHC class II gene and ζ-chain of CD3, or depleting L-arginine which is an essential amino acid for T lymphocyte proliferation [27–31]. Therefore, T lymphocytes are anergy regardless of the specificity of the antigens these cells could encounter.

However, this statement seems less consistent with our results. In our study, we found that MDSCs activated by RDEs accelerated only homologous tumor growth and directly involved in the antigen-specific suppression of CTLs. Willimsky supported this view by statement that tumor-induced MDSCs did not inhibit CD8+ T lymphocyte responses to unrelated antigens in a model of sporadic cancer or other pathogens [32].

Addressing this paradox is based on that these mediators released by MDSCs are very short-lived substances and the function is transient, MDSCs may also suppress T lymphocytes by more stable and prolonged direct cell-to-cell contact [33–35]. That means such contact could induce the antigen specific interaction between MDSCs and T lymphocytes. Of course, ROS and peroxynitrite are also necessary for mediating the factors expressed on the T lymphocytes which render the immunosuppressive to specific antigen. Notably, such medation neither leads to T lymphocyte death, nor prevents the immune response of T lymphocyte to nonspecific antigen.

Other evidence supporting this hypothesis is that MDSCs, acting as antigen-presenting cells, are able to capture tumor-associated antigens and some soluble factors, process and present them to T lymphocytes [36, 37]. This is also be demonstrated in our study that RDEs which enrich with renal specific-antigen and HSP70 could be taken up by MDSCs and drive the activation and expansion of MDSCs. To our knowledge, it plays a crucial role in the antigen-specific tumor immune escape.

Next, Goc et al. reported that mature DCs initiate antigen-specific immunity, resulting in T lymphocyte proliferation and differentiation into helper and effector cells [38]. Our study also showed DCs can be matured using tumor cells lysates and in further trigger CTLs cytotoxicity against autologous tumor cells. DCs are part of normal differentiation from MDSCs. In pathological conditions, such as infections, trauma, sepsis, transpkantation, cancer or some autoimmune diseases, the differentiation of IMCs into mature myeloid cells, such as DCs or macrophages, were partially blocked, resulting in the expansion of MDSCs [39]. Therefore, antigen-specific T lymphocyte immunity may be restricted by this reason in the context of tumor microenvironment.

The antigen-specific immunosuppression induced by MDSCs helps to explain that T lymphocytes in lymphoid organs of tumor-bearing mice or blood of tumor patients can still respond to the stimuli of non-tumor-associated antigens, such as viruses, co-stimulatory molecules, IL-2, CD3−/CD28- specific antibodies [37, 40–42]. Selective removing MDSCs may restores T lymphocytes immunotherapy against tumor, however, targeting of MDSCs is a technical challenge in clinical approaches at present. Here, we investigate potential targets for inducing MDSCs activation.

Although it was demonstrated that RDEs are involved in MDSCs expansion and activation, to date there is also no tool available to prevent exosomes releasing. Mechanistivslly, Both our results and data from another group support the idea that TLRs have a central role in mediating the activation of MDSCs [43], and the activation of MDSCs depended on TLR2/MyD88 pathways. These results suggest that the activation of MDSCs is a fundamental result of the innate response to pathogens expressing TLRs ligand. HSP70 deficiency in RDEs could be an attractive target for inhibiting the activity of MDSCs and tumor progression.

The main limitation of our study is the lack of patients analyzed. Besides, further studies need to determine whether the presence of HSP70 in exosomes could be served as an ideal non-invasive bio-marker for cancer diagnosis and guidance in medication.

## Conclusions

In summary, our study described that MDSCs is a major factor of immune-escape in renal cancer, and the driver of the immunosuppression is antigen-specific. HSP70 enriched in RDEs plays a pivotal role in this process. Unlike previous research most focusing on re-activating the function of T lymphocytes against tumor cells characterized by DNA repair defects and higher neo-antigen loads with increased T lymphocyte infiltration [44, 45], our study described a novel immunotherapeutic strategy. Targeting blockade of either MDSC activity or the direct inhibition of HSP70 can be effective therapeutic strategies and deserve to be clinically evaluated. Once this immunotherapeutic strategy combined with established anticancer treatments, it is highly likely to improve treatment outcome of renal cancers.

## Data Availability

The datasets supporting the conclusions of this article are included within the article and its additional files.

## Abbreviations

MDSCs: Myeloid-derived suppressor cells
RDEs: Renal cancer-derived exosomes
TLR2: Toll like receptor 2
TME: Tumor microenvironment
IMC: Immature myeloid cells
DCs: Dendritic cells
BM: Bone marrow
TDEs: Tumor cell-derived exosomes
HSP: Heat shock protein
MHC: Major histocompatibility complex molecules
MyD88: Myeloid differentiation primary-response gene 88
TLRs: Toll-like receptors
TEM: Transmission electron microscope
DCFDA: 2′,7′-dichlorodihydrofluorescein diacetate
CTLs: Cytotoxic T lymphocytes
CFSE: Carboxyfluoresceindiacetate
PMA: Phrobol-12-myristate-13-acetate
MFI: Median Fluorescence Intensity
LDH: Lactate Dehydrogenase
